# Viral Gastroenteritis Outbreaks in Europe, 1995–2000

**DOI:** 10.3201/eid0901.020184

**Published:** 2003-01

**Authors:** Ben Lopman, Mark Reacher, Yvonne van Duijnhoven, François-Xavier Hanon, David Brown, Marion Koopmans

**Affiliations:** * Public Health Laboratory Service Communicable Disease Surveillance Centre, London, England; † Central Public Health Laboratory, London, England; ‡ National Institute for Public Health and the Environment (RIVM), The Netherlands; § Statens Serum Institute, Copenhagen, Denmark

**Keywords:** surveillance, viral gastroenteritis, infectious intestinal disease, foodborne illness, Norwalk-like virus, Norovirus, research

## Abstract

To gain understanding of surveillance and epidemiology of viral gastroenteritis outbreaks in Europe, we compiled data from 10 surveillance systems in the Foodborne Viruses in Europe network. Established surveillance systems found *Norovirus* to be responsible for >85% (N=3,714) of all nonbacterial outbreaks of gastroenteritis reported from 1995 to 2000. However, the absolute number and population-based rates of viral gastroenteritis outbreaks differed markedly among European surveillance systems. A wide range of estimates of the importance of foodborne transmission were also found. We review these differences within the context of the sources of outbreak surveillance information, clinical definitions, and structures of the outbreak surveillance systems.

Viral pathogens are the most common cause of gastroenteritis in industrialized countries (*1*,*2*). Mead et al. have estimated that of the 38.6 million annual cases of gastroenteritis in the United States, 30.8 million (80%) are the result of viral infections (*3*). Enteric viral pathogens include *Rotovirus A*, *Astrovirus*, adenovirus, and *Sapovirus*, but most viral gastroenteritis infections are caused by *Norovirus* (formerly Norwalk-like viruses) (*1*–*3*). The use of molecular diagnostics including reverse-transcriptase polymerase chain reaction (RT-PCR) and antigen detecting enzyme immunoassays (EIA) (*4*–*20*) have changed researchers’ understanding of the epidemiology of human *Caliciviridae* (including *Norovirus* and *Sapovirus*) (*21*). For example, using RT-PCR assays, Pang et al. showed that caliciviruses were as common a cause of infection as rotaviruses among children <2 years of age (*22*).

In addition, many reports have established the importance of noroviruses as a cause of outbreaks of food- and waterborne illness (*23*–*28*), though estimates of the proportion of infection spread by these modes vary widely: from 14% in England and Wales (*29*) to <40% in the United States (*7*). While person-to-person transmission is probably the mode of infection of most cases, food- and waterborne infections may be of particular importance since these outbreaks have the potential to involve large numbers of people and wide geographic areas and, perhaps, to introduce new variants to an area (*30*).

A research network to study foodborne viruses in Europe was recently funded by the European Union. Through this project, the participant institutes have networked their virologic and epidemiologic surveillance in order to detect transnational outbreaks, elucidate transmission routes, and make international comparisons of the epidemiology of viral gastroenteritis. We chose to study outbreaks rather than community cases because viral gastroenteritis is a very common infection (*1*); therefore, enumeration of epidemics (or outbreaks) may be more practical and useful since individual cases are poorly reported (*31*). International comparisons of surveillance data are difficult because criteria for effective surveillance customarily varies across borders (*32*).

The objective of this survey was to capture information on the structure of outbreak surveillance in each country (including sources of data and definitions employed) and to gain estimates of the frequency of outbreaks, as well as to compare the setting of outbreaks, the importance of foodborne transmission, and the use of characterization techniques. We present surveillance data from viral gastroenteritis outbreaks from 1995 to 2000 collected by participant European countries. These data provide baseline information for future harmonization and comparison efforts.

## Methods

A questionnaire was sent by e-mail to the project leaders of the 13 participant institutions (from 10 countries) in the Foodborne Viruses in Europe group. The questionnaire, administered in English, was developed and completed in collaboration with research and medical virologists and epidemiologists working in viral gastroenteritis surveillance. General information on surveillance systems (including sources of data, estimate of national population under surveillance, definition of a viral gastroenteritis outbreak, and number of such outbreaks investigated) was collected for the period 1995–2000. More detailed epidemiologic data (setting, mode of transmission, and implicated food vehicles) were collected from outbreaks that occurred in 2000. Contributors were sent a summary report and asked to confirm that the data presented accurately represented their surveillance.

## Results

### Data Sources of Surveillance Systems

One completed survey questionnaire was received from all 10 countries. A range of sources contributed data on viral gastroenteritis outbreaks for European surveillance systems (Table 1), including diagnostic reference laboratories, local public health staff, food inspectorates, and physicians. We derive our data from routine surveillance except for Germany, where systematic national surveillance was not operational during the survey period. German data were collected from laboratories that performed RT-PCR diagnostics in the surveyed period. The same applies to the Netherlands, Finland, and Sweden, although the collaborating centers in these countries run the sole reference laboratory service.

**Table 1:** Sources of information of viral gastroenteritis surveillance systems in the Foodborne Viruses in Europe network

Country	Sources of outbreak data				
	Diagnostic microbiology laboratory	Food safety inspectorate	Physician/ patient reports	Local/regional public health authority	Type of outbreaks reported
Denmark		Yes	Yes	Yes	Food/waterborne
France	Yes			Yes	Food/waterborne
England and Wales				Yes	All
Italy	Yes				All
Finland	Yes^a^				All
Sweden	Yes				All
Germany	Yes			Yes^b^	All
Slovenia	Yes			Yes	All
Spain	Yes			Yes	All
the Netherlands^c^	Yes	Yes	Yes	Yes	All^d^

^a^Participant is sole laboratory performing viral testing, and coordination is conducted at National Public Health Laboratory and National Food Administration. ^b^*Norovirus* became a reported disease in January 2000. From 1997 to 2000, reports from local health departments were collected unsystematically. ^c^Dutch national data were collected from three systems: notification system, food safety inspectorate, and laboratory-based system (from diagnostic microbiology laboratories, local/regional public health authorities, physician/patient reports, and other institutions in which outbreaks occurred). ^d^Foodborne only for systems 1 and 2.

### Outbreak Definition and Geographic Coverage of Surveillance Systems

All surveillance systems reported data collected on outbreaks from the whole population of their respective countries except for Italy, where a small geographically convenient sample of approximately 1% of the population was covered by surveillance (Table 2). Both the criteria and the use of outbreak definitions differed among the surveillance systems (Table 2). Some systems collected information only on incidents that met a specific definition; other systems collected information on all incidents and then applied definitions retrospectively for analysis. Some surveillance systems required laboratory confirmation to attribute an outbreak to an enteric viral pathogen. Among systems requiring laboratory confirmation, a range of stringency existed from at least one positive sample (England and Wales) to half of all stools positive for virus (Finland and the Netherlands).

**Table 2:** National coverage and use of clinical definitions for viral gastroenteritis by European surveillance systems

Country	National coverage %^a^	Definition of viral gastroenteritis outbreak^b^	Laboratory confirmation required	Outbreak definition applied		
				As entry criteria in database	Retrospectively for analysis	
Denmark	100	Kaplan’s, shellfish		Always	Always	
England and Wales	100	General	Yes	Always	Never	
Finland	100	Clinical	Yes	Always	Never	
France	100	Clinical, shellfish		Always	Never	
Germany						
Italy	1	Clinical		Always	Always	
Slovenia	100	Clinical		Sometimes	Sometimes	
Spain	100	General	Yes	Always	Sometimes	
Sweden	100	Kaplan’s, clinical	Yes	Always	Sometimes	
the Netherlands	100	System 1: clinical System 2: Kaplan’s System 3: clinical	Yes Yes	Sometimes	Always	

^a^Refers to geographic coverage by surveillance, not completeness of reporting. ^b^Kaplan’s, Kaplan’s criteria for recognition of *Norovirus* outbreaks (*33*); clinical, clinical criteria (different from Kaplan’s) specifying that cases must be clustered in time and place; general, general definition used for all outbreaks of gastroenteritis with laboratory confirmation required to attribute outbreak to viral pathogen; shellfish, specific criteria used for identifying shellfish outbreaks.

### Outbreaks Investigated

Outbreak reports were available from the entire surveyed period (1995–2000) from a few countries: England and Wales, Slovenia, Spain, the Netherlands, and Sweden. The overall numbers of outbreaks investigated ranged from 2 in Italy to 1,643 in England and Wales (Table 3).

**Table 3:** Reported outbreaks of viral gastroenteritis, European surveillance, 1995–2000

	1995	1996	1997	1998	1999	2000			Total
						All viral organisms	*Rotavirus* ^a^	*Norovirus* ^a^	1995–2000
Denmark				9	11	17	0	17 (100)	37
England and Wales	392	352	151	219	239	290	13 (4)	273 (96)	1,643
Finland			5	27	35	58	1 (2)	56 (97)	125
France	4	9	7	8	19	28	1 (14)^b^	5 (71)^b^	43
Germany			1	53	145	227	0	227(100)	426
Italy	0	0	0	0	0	2	0	2 (100)	2
Slovenia	8	6	8	4	5	14	8 (57)	6 (43)	45
Spain	37	24	25	29	66	55	6 (43)^c^	8 (57)^c^	236
Sweden	81	130	130	130^d^	190^d^	195^d^		190 (97)	856
the Netherlands	25	69	54	36	58	59	5 (13)^e^	32 (84)^e^	301

^a^Number of outbreaks attributed to organism (percentage of year 2000 outbreaks). ^b^Based on seven laboratory-confirmed viral outbreaks. ^c^Based on 14 laboratory-confirmed viral outbreaks. ^d^Approximate figures. ^e^Based on 38 laboratory-confirmed viral outbreaks.

**Figure 1:**
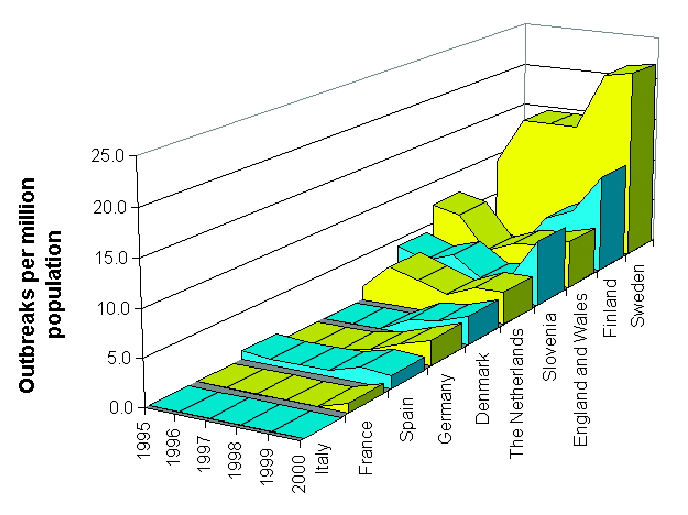
Viral gastroenteritis outbreak rates, European surveillance, 1995–2000. Rates based on year 2000 national population estimates.

National outbreak reporting rates for each country were calculated by dividing annual outbreaks by national population (Figure 1). Rates in Sweden (9–22 outbreaks/million in population) were markedly higher than in any other country. In most countries, approximately 3–7 outbreaks per million population were ascertained annually. Since 1997, outbreak reporting rates have been increasing in most countries.

### Completeness of Basic Epidemiologic Data

**Figure 2:**
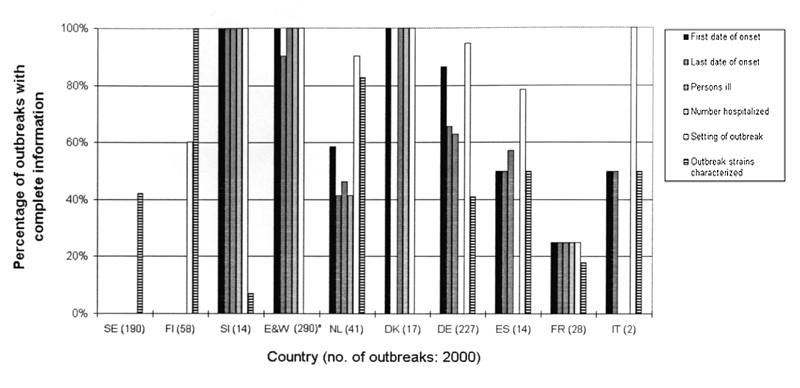
Completeness of epidemiologic and viral characterization information on viral gastroenteritis outbreaks, European surveillance, 2000. SE, Sweden; FI, Finland; SI, Slovenia; E&W, England and Wales; NL, the Netherlands; DK, Denmark; DE, Germany; ES, Spain; FR, France; IT, Italy. *Approximately 500 outbreaks strains were characterized in the United Kingdom, but typing is not linked to epidemiologic data.

Participants were asked how many of the outbreaks reports from the year 2000 included details on first date of onset, last date of onset, number of persons ill, number of persons hospitalized, number of related deaths, and setting of the outbreak. Completeness of these data differed substantially between countries: none of the data were available from Sweden, whereas data were almost 100% complete for all categories in England and Wales, Denmark, and Slovenia (Figure 2).

### Setting of Outbreaks

**Figure 3:**
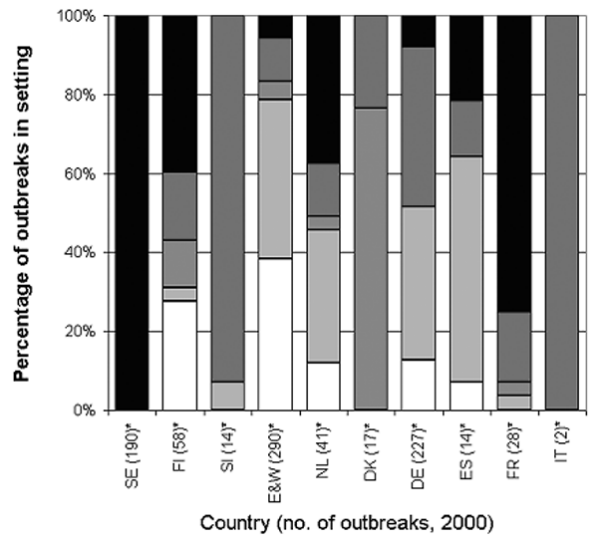
Setting of viral gastroenteritis outbreaks, European surveillance, 2000. SE, Sweden; FI, Finland; SI, Slovenia; E&W, England and Wales; NL, the Netherlands; DK, Denmark; DE, Germany; ES, Spain; FR, France; IT, Italy. *Includes restaurants, cafes, public bars, mobile vendors, canteens, and catered events.

The settings where reported outbreaks occurred differed substantially by country (Figure 3). In England and Wales, Spain, and the Netherlands, most reported outbreaks occurred in hospitals and residential homes (78%, 64%, and 66%, respectively), whereas in Denmark, 13 (76%) of 17 reported outbreaks occurred in food outlets. In Denmark, surveillance is done by the Food Safety Inspectorate, which collects reports of suspected foodborne outbreaks only. The Inspectorate is not informed of person-to-person spread outbreaks, which are more commonly seen in residential institutions and hospitals.

In Slovenia, the majority of reported outbreaks occurred in day-care centers (10/14; 71%), and in France, most reported outbreaks occurred in private houses (7/9; 78%). In France, reporting was recommended only for large outbreaks or if oysters, an item commonly consumed in French households, were the suspected vehicle of infection.

### Food and Water as Sources of Outbreaks

Among countries conducting broad-based outbreak surveillance, the following proportions of viral gastroenteritis outbreaks were reported to be associated with food- or waterborne transmission: Finland (24%), the Netherlands (17%), Slovenia (14%), Spain (7%), and England and Wales (7%) (Table 4). Very rarely was laboratory evidence (detection of the same organism in the vehicle and stool specimens) or statistical evidence (case-control or cohort) available that demonstrated the association of the vehicle with illness. During the survey period, Danish and French surveillance almost exclusively focused on outbreaks transmitted through food and water. Therefore, estimates of the proportion of food and water transmission from these countries cannot be compared to the general estimates in other countries.

**Table 4:** Foodborne transmission and supporting evidence of implicated food vehicles, European surveillance, 2000

Country	Total outbreaks	Food/waterborne outbreaks (%)	Evidence		
			Laboratory^a^	Statistical^b^	
Denmark	17	16 (94)	1	0	
England and Wales	290	20 (7)	1	4	
Finland	58	14 (24)	0	0	
France	28	28 (100)	2	1	
Germany	227				
Italy	2	0			
Slovenia	14	2 (14)	0	0	
Spain	14	1 (7)	0	1	
Sweden	190				
the Netherlands	41	7 (17)	0	2	

^a^Same organism found in stool specimen and food vehicle. ^b^Statistically significant result from cohort or case-control study.

### Molecular Characterization Techniques

Different molecular techniques were used by participating institutes to characterize virus from outbreaks in 2000. Reverse line blot was used in the Netherlands and Spain, and the heteroduplex mobility assay was used in England and Wales. Sequence analysis was performed in England and Wales, Finland, France, Italy, Germany, Spain, and the Netherlands; EIA were used in England and Wales, and a microplate hybridization technique was used in Finland.

## Discussion

Viral gastroenteritis infection, typically a self-limiting condition of short duration in humans, is extremely common and associated with relatively low deaths. Surveillance of outbreaks of this infection, rather than individual cases, may be more appropriate. In our review of the surveillance for this infection in Europe, we found variations in the organizations conducting surveillance, the surveillance definition of a viral gastroenteritis outbreak, the populations under surveillance, and the completeness of descriptive and analytical epidemiologic and diagnostic information.

Researchers comparing surveillance information at an international level should consider the outputs of surveillance, as well as the influence of methodology and structure of surveillance on these outputs. Surveillance for viral gastroenteritis in Europe is poorly developed; systems vary in their sources of data, definitions, and use of diagnostic techniques. These differences are reflected in the wide range of numbers of outbreaks, population-based rates, and epidemiologic patterns observed across Europe. Nonetheless, our comparison of this surveillance data was an informative exercise because international epidemiologic databases of viral gastroenteritis infections have not been developed. In many of the countries included in the Foodborne Viruses in Europe network, viral gastroenteritis has not been considered a priority, and these countries do not have a well-developed surveillance system. This inventory of surveillance data will aid in the development of a more consistent and complete surveillance across Europe.

These data clearly show that both the absolute number and the population-based rates of viral gastroenteritis outbreaks differ substantially between European surveillance systems. From 1995 to 2000, 1,643 outbreaks of viral gastroenteritis were investigated by the Public Health Laboratory Service in England and Wales, but the outbreak rates (number of outbreaks/population) were highest in Sweden for every surveyed year. Some variation in these figures occurred because a number of the surveillance systems required laboratory confirmation while others did not (Table 2). However, the criteria suggested by Kaplan et al. to recognize an outbreak of viral etiology is widely used and is generally accepted as an effective clinical tool in the absence of diagnostic information (*33*). Interestingly, surveillance systems with the most stringent outbreak criteria, including laboratory confirmation of outbreaks (England and Wales, Finland, and Sweden) ascertained the most outbreaks, likely because surveillance in these countries is more developed and integrated better with reporting bodies.

However, even the surveillance systems with the highest figures greatly underascertain viral gastroenteritis. A study of infectious intestinal disease in England and Wales estimated that only 1/300–1,500 cases of *Norovirus* gastroenteritis are reported to national surveillance (*34*). For a case to be ascertained by national surveillance, patients must be examined by their primary-care doctor, a specimen must be taken and submitted for laboratory testing, the test must be positive (the amount of false negatives will depend on the diagnostic technique), and the surveillance unit must be notified. Ascertaining outbreaks requires an additional step in which investigators must recognize epidemiologic links between cases. While this chain of events will differ from country to country, the principle of underascertainment affects all surveillance. However, outbreak recognition and investigation will, through case finding, lead to better ascertainment of persons affected in outbreaks.

Although most surveillance systems may be designed for national coverage, reports were incomplete to a varying degree. Ascertained outbreaks varied geographically and were incomplete, as demonstrated by the large variation in reported outbreaks (Table 3).

This survey found that the great majority of European viral outbreaks could be attributed to *Norovirus*. In Denmark, England and Wales, Finland, France, and Sweden, >95% of nonbacterial outbreaks were attributed to noroviruses as were 84% of outbreaks in the Netherlands. The relative number of infections from noroviruses was lower in Slovenia (43%) and Spain (57%), although these estimates are based on a small number of outbreaks (n=14 for both). These figures are consistent with previous reports that *Norovirus* could be detected in 91% of all nonbacterial infectious intestinal disease outbreaks in the Netherlands (*9*) and 89% of such outbreaks in Sweden (*35*). Similarly, Fankhauser et al. found *Norovirus* responsible for 96% of nonbacterial outbreaks in the United States (*7*).

Estimates of the importance of foodborne transmission also varied widely in this survey. Foods were implicated as the vehicle of transmission in 16 (94%) of 17 outbreaks in Denmark and 28 (100%) of 28 outbreaks in France because surveillance systems in these countries were designed to detect foodborne disease. In countries with more general outbreak data, estimates of foodborne transmission were lower: 7 (17%) of 41 in the Netherlands, 14 (24%) of 58 in Finland, and 20 (7%) of 290 in England and Wales, although laboratory and statistical evidence of association with food or water was scant.

The settings of outbreaks also reflected the proportion of reported outbreaks that were ascertained to be foodborne. For example, in Denmark, 75% of all reported outbreaks were set in food outlets. In Spain, the Netherlands, and England and Wales, most reported outbreaks occurred in residential homes and hospitals, with only a small fraction occurring in food outlets.

In Finland, the National Public Health Laboratory is the only facility in the country testing for *Norovirus* and, therefore, is aware of all such investigations. Most other surveillance systems receive data on outbreaks from a number of sources including local public health authorities, other diagnostic laboratories, and physicians. Surveillance in Denmark is anomalous in that only outbreaks from the national food inspection service are reported, which, in conjunction with the special mention of shellfish in the definition of an outbreak, explains the preponderance of food-related outbreaks in Danish surveillance. Such diversity in data sources and definitions may also explain the differences in estimates among other countries, including those external to the Foodborne Viruses in Europe network. Based on data from 90 outbreaks, Fankhauser et al. estimated that 47% of *Norovirus* outbreaks in the United States were spread by food (*7*). This estimate, derived from local and state health department reports, may be affected by reporting bias or may truly reflect different epidemiologic patterns of viral gastroenteritis outbreaks compared to those seen in European countries. Factors that might affect the relative amount of foodborne transmission of *Norovirus* are the virologic quality of food, food-handling guidelines, and infection control practice in health-care settings (*36*).

DNA sequencing of PCR amplicons, used to characterize outbreak strains by laboratories in England and Wales, Finland, France, Italy, Germany, Spain, and the Netherlands, yields the most virologic information, although this technique is a labor-intensive procedure. The heteroduplex mobility assay (*37*), as well as an EIA based on one of the *Norovirus* genotypes (Grimsby virus), was used by the laboratory in England and Wales. Reverse line blot (*38*) was used in the Netherlands and Spain in 2000 and has since been adopted by a number of the other collaborating institutes to characterize *Norovirus*. The use of the heteroduplex mobility assay (*37*), reverse line blot (*38*), and sequencing to characterize virus has demonstrated the considerable and dynamic genetic diversity of human *Caliciviridae* (*39*). The use of such techniques by a wider group may demonstrate important differences in molecular epidemiology between countries and may detect the introduction of a novel strain to an area (*40*) by tracking and linking outbreaks over wide geographic areas.

In the retrospective survey presented here, determining whether differences in frequency, setting, and importance of foodborne transmission are real or artifacts caused by international variation in surveillance system design was difficult. Timely collection of information for case-control or cohort studies and development of tests for virus detection in food are needed to advance understanding of the extent of foodborne transmission of *Norovirus*. Success of the Foodborne Viruses in Europe network will depend on the ability to compare both virologic and epidemiologic data. Protocols for harmonizing the characterization of noroviruses and an outbreak questionnaire with a minimum dataset have been defined. While data collection will be harmonized, information will be obtained from an international group whose range of perspectives will yield different interpretations of epidemiologic events. The timely feedback of surveillance data to participants is an essential step in the cycle of continued improvement of a surveillance system (*41*) that we have made possible through this European Union–funded network. In addition to describing the current state of viral gastroenteritis surveillance in Europe, this report will act as a baseline to interpret prospective outcomes of the Foodborne Viruses in Europe network.

Foodborne Viruses in Europe is funded by the European Commission, Directorate General Research under the Quality of Life and Management of Living Resources- QLK1-CT- 1999-00594.

Mr. Lopman works as an epidemiologist at the Gastrointestinal Diseases Division of the Public Health Laboratory Communicable Disease Surveillance Centre. He coordinates the epidemiologic surveillance for the Foodborne Viruses in Europe consortium.
